# The Potential for Xanthine Oxidase Inhibition in the Prevention and Treatment of Cardiovascular and Cerebrovascular Disease

**DOI:** 10.1155/2009/282059

**Published:** 2009-11-04

**Authors:** Peter Higgins, Jesse Dawson, Matthew Walters

**Affiliations:** Division of Cardiovascular and Medical Sciences, Faculty of Medicine, University of Glasgow, Glasgow G11 6NT, UK

## Abstract

There is a now a wealth of epidemiological, animal, and clinical data to suggest the benefits of uric acid reduction and hxanthine oxidase inhibition in prevention of vascular disease. This review discusses the available epidemiological, preclinical, and clinical data and considers arguments for and against a role for serum uric acid in common cardiovascular disorders. It concludes that large scale trials with clinical endpoints are justified to address this important question and to define whether use of drugs such as allopurinol should be a routine part of preventative strategies.

## 1. Introduction

Xanthine oxidase inhibitors are typically used in the treatment of gout and nephropathy and renal stone diseases linked to hyperuricaemia. There has been recent interest in the potential benefit of these agents in the prevention of vascular disease, including those affecting the cerebrovasculature. This interest has been driven by emerging evidence suggesting a role for serum uric acid in the development of cardiovascular disease and because xanthine oxidase inhibition may yield ancillary benefits over uric acid reduction; the enzyme is an important source of oxidative stress in the vasculature.

In this review we summarise available epidemiological, preclinical, and clinical data and consider arguments for and against a role for serum uric acid in common cardiovascular and cerebrovascular disorders. Against this background we also discuss the potential benefit of xanthine oxidase inhibition as a vascular preventative strategy.

## 2. Uric Acid and Xanthine Oxidase?

Uric acid is a breakdown product of ingested and endogenously synthesised purines. DNA and RNA are degraded into purine nucleotides and bases, which are then metabolised, via the action of xanthine oxidase, to xanthine and then uric acid. Xanthine oxidase is from the molybendum iron-sulfur flavin hydroxylase group of enzymes and is found predominantly in the liver and gastrointestinal tract but also in the kidney and brain. Interestingly, it is also found throughout the cardiovascular system [1], and endothelial bound forms have been described. Expression of these has been shown to increase in ischaemia and in response to increased levels of proinflammatory cytokines [2]. While the major role of xanthine oxidase is conversion of hypoxanthine and xanthine to uric acid, an interconvertible form, xanthine dehydrogenase, also exists and is responsible for conversion of NAD^+^ to NADH [3]. The action of these enzymes yields hydroxyl free radicals and hydrogen peroxide which can add to or initiate oxidative stress [4].

In most mammals, the enzyme uricase further oxidizes uric acid to allantoin, which is then excreted in the urine. In humans and higher primates however, uric acid undergoes no further metabolism and homeostasis relies upon excretion of uric acid, predominantly via the kidneys. Levels are thus higher, and humans also have the ability to reabsorb uric acid in the proximal tubule, via the action of a urate transporter [21]. While uric acid levels can be increased by rare enzymatic defects, states of high cell turnover and alcohol ingestion [22, 23], the majority of cases of elevated serum uric acid, and the population distribution of levels result from differences in renal excretion.

The reason for this difference in humans is unclear and whether this was a protective and beneficial change will be discussed in more detail later [24].

## 3. Is Uric Acid a Risk Factor—Epidemiology?

Most epidemiological studies associate increasing serum uric acid with increased cardiovascular event rate and mortality in those with known or elevated risk of vascular disease and amongst healthy volunteers. These data have been thoroughly reviewed elsewhere [25] but are summarised in Table 1. Of interest, the relationship with stroke rate and mortality has often been less clear. For example, in those with hypertension, many studies have not specifically evaluated stroke mortality or have been limited by low observed stroke rates [16, 26]. As an example, one Chinese study has shown a strong association [15] while others have not [13, 17]. An association has been seen with stroke risk in patients with diabetes [9] and in a large observational cohort of patients aged >55 years of age, although the association was less prominent in hypertensive individuals [27]. A recent analysis of hypertensive individuals, in which there were 354 stroke deaths (the highest number in any study to date), showed that there was indeed a link between increasing serum uric acid and stroke mortality but that this relationship was J-shaped, unlike the significant linear relationships seen between uric acid and total vascular and coronary mortality [28].

There is also controversy concerning the impact of serum uric acid on outcome in the acute period after stroke where antioxidant activity could be considered highly beneficial, as discussed later. In a large study of those with acute stroke, increasing serum uric acid levels was associated with a reduced likelihood of favourable outcome at 90 days (odds ratio 0.78, 95% CI 0.67–0.91 per additional 0.1 mmol/L uric acid) and an increased risk of recurrent vascular events [5]. This association was more prominent in those with diabetes [10]. Others have suggested increased risk of early clinical deterioration following ischaemic stroke [6] in those with increased serum uric acid level. However, in contrast, a further study reported conflicting results: in 800 patients with acute ischaemic stroke, increasing uric acid levels was associated with a good outcome (odds ratio 1.12, 95% CI 1–1.25 per additional mg/dL uric acid) at seven days [8]. A recent analysis from our unit however, found that increasing uric acid levels did link with increased odds of poor outcome (but not in an independent fashion), and there was no evidence that increasing serum uric acid conveyed protection to the ischaemic brain [7] and others have recently shown increased risk of death early after stroke [29].

## 4. Uric Acid Is Harmful

Uric acid crystals clearly have the potential to induce inflammation given their role in pathogenesis of gout. Serum uric acid levels have also been linked to levels of proinflammatory cytokines and may have a role in perpetuating the inflammatory response that characterises atherosclerosis. Uric acid may also increase oxygenation of LDL [30], and uric acid crystals have also have been shown to stimulate release of the platelet constituents serotonin, ATP and ADP [31]. 

Uric acid has been shown to stimulate rat vascular smooth muscle production in vitro [32], and it has also been shown to link with endothelial dysfunction in those with hypertension. Perhaps most importantly, uric acid has a putative role in the development of hypertension [33–35] via effects on nitric oxide production in the macula densa. Studies have shown that uric acid reduction with allopurinol can improve blood pressure in adolescents with newly diagnosed hypertension as discussed later.

## 5. Uric Acid Is Protective

It is clear that our life span has increased markedly during evolution and this may in part reflect a reduction in early cancer rates compared to other mammalian species. It has been argued that this is due to evolution of more effective antioxidant mechanisms and that higher levels of serum uric acid are a key component of such mechanisms [24]. Given that uric acid is the most abundant antioxidant in plasma, it is feasible that this change in uric acid metabolism has been key to our prolonged survival. This is supported by an elegant series of experiments suggesting that uric acid does indeed protect against oxidative damage and is as effective as ascorbic acid [24]. Paradoxically, however, focus has now shifted to an accusatory role for uric acid in disease.

Uric acid has been shown to have antioxidant activity in humans, and the rising levels that have accompanied human evolution may thus have been beneficial and have increased longevity. This hypothesis is further supported by data showing that in healthy human volunteers, uric acid administration increases total serum antioxidant capacity [36] and reduces oxidative stress associated with exercise.

There is also evidence that serum uric acid levels increase after an ischaemic insult and many argue that elevated serum uric acid represents a physiological and protective response to oxidative stress and acute vascular insults [37, 38]. This hypothesis is supported by the previously mentioned study where increasing serum uric acid levels linked with a good clinical outcome [8] and other surrogate markers of outcome. In a rat model of cerebral ischaemia, brain uric acid levels have been shown to increase [39, 40], and in a transient ischaemia model, infusion of uric acid led to a reduction of infarct volume and improved behavioural outcome [40] suggesting therapeutic potential for infusion of serum uric acid. Similar findings were demonstrated in models of traumatic brain injury and multiple sclerosis [41, 42]. Data from twin studies also suggest that uric acid may be beneficial for the brain; levels of uric acid in sufferers of multiple sclerosis have been found to be lower than in healthy siblings [43]. 

Recently, the therapeutic potential of uric acid administration after stroke has been explored in humans. In a small study of individuals treated with intravenous thrombolytic therapy, intravenous infusion of uric acid in the early period after ischaemic stroke reduced markers of oxidative stress [44].

## 6. Alternative Explanations—The Innocent Bystander

It is argued that the apparent link between serum uric acid and disease simply reflects the presence of other risk factors. For example, serum uric acid is known to link and cluster with other risk factors, and it may be that atherosclerosis itself or increased oxidative stress leads to increased serum uric acid hence the apparent association.

Worsening renal function is associated with both increased serum uric acid levels and increased burden of cardiovascular disease, although most epidemiological studies have adequately attempted to adjust for renal impairment. High levels of serum uric acid link with presence of obesity, adverse lipid profiles, including low HDL levels, and insulin resistance [45–47]. As mentioned there is an association with blood pressure, and uric acid has been implicated in the development of hypertension [48, 49] and use of allopurinol has led to falls in blood pressure in adolescents with newly diagnosed hypertension [49].

It is also possible that higher levels of uric acid may reflect higher levels of xanthine oxidase activity and oxidative stress. The action of xanthine oxidase leads to generation of superoxide anions and is one of the principle sources of reactive oxygen species (ROS) in the human vasculature [50, 51]. The molecular effects and importance of ROS in cardiovascular disease has already been extensively reviewed [52–55]. Traditionally, xanthine oxidase has been considered significantly less important than NADPH oxidase as a source of oxidative stress in the vasculature. Emerging evidence suggests that the reverse may be more representative of the in vivo situation. Studies suggest that xanthine oxidase activity is greatly increased in those with heart failure and in response to ischaemia [1, 2]. On a practical level, potential for clinical relevance is much greater; a licensed and commonly used inhibitor of xanthine oxidase exists, which of course is not the case for NADPH oxidase.

Thus, regardless of whether serum uric acid itself is harmful, it could still prove to be a powerful marker of a high-risk disease state by helping identify those with increased xanthine oxidase activity which, via oxidative stress, may directly contribute to the development of atherosclerotic disorders and vascular events.

## 7. Does Lowering Serum Uric Acid Modify Cardiovascular Risk (with Agents Other than Xanthine Oxidase Inhibitors)?

There are no adequately powered clinical endpoint trials of uric acid lowering strategies. However, three drugs known to reduce cardiovascular mortality have been shown to reduce serum uric acid, which, hypothetically, may explain some of their beneficial effect.

Fenofibrate lowers triglyceride and total and LDL cholesterol levels and increases HDL cholesterol [56]. It also reduces serum uric acid level (via increased renal excretion) by as much as 46% in healthy volunteers and hypertensive and diabetic patients and has an effect additive to other urate lowering therapies [57–59]. Losartan is an angiotensin II receptor antagonist known to reduce serum uric acid levels by as much as 30% [60], via increased renal uric acid excretion [61]. Nearly a third of the modest relative risk reduction seen with losartan use in the LIFE study [62] has been attributed to its effect on serum uric acid [63]. As a further example, atorvastatin has been shown to reduce serum uric acid (by approximately 8%), even after adjustment for risk factors including change in renal function. Each 60 *μ*mol/L reduction in serum uric acid following atorvastatin use was associated with a reduction in vascular event rates (HR 0.76, 95% CI 0.62–0.89) [64].

In addition to these large trials, the effect of Probenecid (a uricosuric agent with no effect on xanthine oxidase) has been studied in a a group with heart failure. In a randomised crossover design, Probenecid 500 mg bid for three weeks reduced uric acid (to 0.25 mmol/L) in comparison to placebo (where it remained increased at 0.44 mmol/L). This reduction was similar to that observed with allopurinol 300 mg od over three weeks but no improvement in endothelial function was seen (whereas it was with allopurinol) suggesting that the mechanism of benefit of allopurinol is uric acid-independent [65].

## 8. Does Xanthine Oxidase Inhibition Modify Cardiovascular Risk?

Recent relevant clinical research has focussed on the use of the xanthine oxidase inhibitors allopurinol and oxypurinol in the prevention of cardiovascular diseases. Allopurinol is a structural analogue of hypoxanthine and is rapidly metabolised to oxypurinol, which functions similarly. They preferentially bind to xanthine oxidase thereby inhibiting its activity [66]. This will lower uric acid levels but also xanthine oxidase mediated free radical production. Furthermore, there is evidence that the drug has a direct scavenging effect on free radicals.

The effect of xanthine oxidase inhibition on measures of endothelial and cardiovascular function has been tested in small studies of those with heart failure, coronary artery disease, stroke, diabetes, hypertension, hypercholesterolaemia, smokers, elevated 10-year cardiovascular risk, metabolic syndrome, COPD, sleep apnoea, the elderly, and in those with chronic liver disease [65, 67–100]. The findings of these are summarised below in order of the endpoints assessed.

### 8.1. Endothelial Function and Oxidative Stress

Improvement in endothelial function has been observed following xanthine oxidase inhibition in patients with heart failure [65, 69, 70], coronary artery disease [75, 90], diabetes [78], hypercholesterolaemia [81], smokers [83, 95], high overall cardiovascular risk [84], metabolic syndrome [85], obstructive sleep apnoea [86], and stroke [87]. 

In some studies benefit has been restricted to those with hyperuricaemic [69, 84]. Also, some studies have found no improvement following xanthine oxidase inhibition in healthy subjects [96], patients with hypertension [81], and a group with hypercholesterolaemia [82]. Further, it is a consistent finding that those included in healthy control groups show no benefit from xanthine oxidase inhibition [78, 81, 83, 95, 96].

Levels of oxidative stress in the circulation have been shown to reduce in response to xanthine oxidase inhibition in subjects with heart failure [69, 70], diabetes [79], metabolic syndrome [85], obstructive sleep apnoea [86], coronary artery disease [88, 90], chronic obstructive pulmonary disease [96, 97], and liver disease [99]. Markers of oxidative stress did not reduce in some studies of healthy volunteers [96] and in those with diabetes [80], and again, in healthy control arms of many studies, no benefit was seen.

These differing results may be accounted for by differences in study design (in particular small sample size and baseline subject characteristics). A further consideration is the potential for benefit in those who do not have significant impairment of vascular function. As mentioned, data are less compelling in those with only vascular risk factors but no established disease and in healthy control arms. Xanthine oxidase may have a limited role in determining endothelial function in those with a “healthy” vascular system, and indeed, evidence exists to suggest that enzyme activity is increased during stresses such as ischaemia [1]. Furthermore, xanthine oxidase may not be universally integral to endothelial dysfunction in all cardiovascular disease conditions.

Another intriguing finding from some studies is that benefit may be limited to those with hyperuricaemia only, perhaps because hyperuricaemia reflects pathological levels of enzyme activity.

### 8.2. Haemodynamic/Cardiac Outcomes

Improvement in blood pressure has been reported following xanthine oxidase inhibition [91, 93, 94].

Improvement in cardiac function, including left ventricular ejection fraction [72, 76, 88], cardiac index [88], end-systolic volume [76], and myocardial efficiency [67] has been documented in those with cardiac failure, although no difference in heart rate, dysrhythmia count [68] or exercise capacity was found [71–73]. A reduction in “infarct extension” has been reported in an allopurinol treatment group in the acute coronary syndrome setting, though methodological considerations make interpretation of this finding difficult [89].

Long-term outcomes in response to xanthine oxidase inhibition have been assessed in those with heart failure. A medium-sized prospective study identified no overall difference following oxypurinol treatment upon a combined outcome of heart failure-related mortality/morbidity/quality of life but did suggest potential benefit amongst hyperuricaemic patients [73]. One large retrospective analysis suggested a protective effect with high-dose allopurinol over low-dose treatment [100]. Intriguingly the study found that low-dose allopurinol was associated with a poorer outcome compared with no treatment at all. The retrospective study design does not permit a definitive explanation for this paradox but perhaps lends further weight to the concept that the interplay between uric acid level and xanthine oxidase activity and vascular health is complex and achieving optimal levels of each may be challenging.

### 8.3. Humoral/Inflammatory Indices

Some studies have shown an improvement in renal function [92, 94] following allopurinol use. C reactive protein, a surrogate marker for chronic inflammation, has also been found to reduce in response to treatment [94], though this finding has not been reproduced in other studies [65, 71, 77, 85]. The above noted improvements in cardiac physiology have been supplemented by the finding that brain natriuretic peptide, a prognostic indicator in heart failure, reduced in one study [71]. One study identified a reduction in cholesterol with allopurinol versus placebo, *P* < .05 [71], though this finding is yet to be reproduced.

In those with recent stroke, we have shown that use of allopurinol attenuates the rise in inflammatory markers seen after stroke [77] and also that it improves basal levels of cerebrovascular nitric oxide in those with diabetes [101]. In this study, the response to infusion of NG-Monomethyl-L-Arginine (L-NMMA) was assessed; in the cerebral vasculature, L-NMMA reduces cerebral blood flow through restriction of NO activity, and the higher the basal NO activity, the larger the effect. Allopurinol enhanced this effect implying that it improves basal levels of NO activity and the response improved towards levels seen in healthy volunteers.

However, we must acknowledge that results from the largest study to date were disappointing. In the OPT-CHF trial [73], 405 participants with heart failure were randomised to receive either oxypurinol 600 mg or placebo for 6 months. The primary endpoint was defined as a change in clinical status based upon changes in a variety of clinical parameters including mortality and common measures of heart failure severity. There was no difference in the proportion of patients who improved or worsened between treatment groups (43.3% improved with allopurinol compared to 45%) with placebo while 32% and 35.6% remained the same and 24.6% and 19.3% worsened (*P* = .42). However, posthoc analyses suggested that in those with elevated serum uric acid, oxypurinol improved clinical status, whereas the opposite occurred in those with lower uric acid levels. In the oxypurinol cohort as a whole, those who improved had significantly greater reductions in serum uric acid levels than those who worsened. These findings are difficult to interpret. On one hand the posthoc analyses suggest benefit in those with high uric acid levels and that the greater the fall following oxypurinol in such patients, the better the outcome; they also raise the possibility that oxypurinol can cause harm in some.

## 9. Hypothesis—Is Uric Acid Good, Bad, or Indifferent?

The epidemiological evidence does suggest that elevated serum uric acid links with increased incidence and severity of a variety of cardiovascular diseases; yet we know that it has antioxidant properties, and small preclinical and clinical studies suggest that serum uric acid may be neuroprotective. However, data do exist to support detrimental and prothrombotic effects of uric acid on platelet and endothelial function, and an ever growing number of clinical studies suggest that uric acid lowering strategies do reduce vascular risk, although for the most part the evidence concerns use of allopurinol which may have other beneficial effects; xanthine oxidase mediated oxidative stress is likely to have a significant role in the development of atherosclerosis.

It is important to note that these hypotheses are not mutually exclusive. Firstly, increased local tissue levels of uric acid in ischaemia and brain injury may reflect levels of oxidative stress and xanthine oxidase activity and thus the mechanism of harm and not an innate protective response. Essentially, the substance itself may well have antioxidant properties but its generation and associated superoxide anion production may be of much greater significance and detriment in the longer term. Thus, the antioxidant properties of uric acid could well be harnessed to improve clinical outcomes in the acute phase of cardiovascular and neurological illness. Furthermore, the measurement of uric acid levels in those at risk of disease may identify those at high risk who may benefit from treatments such as allopurinol, either because of uric acid itself or because of xanthine oxidase activity and oxidative stress. Perhaps data from the OPT-CHF study support these seemingly competing views; benefit from uric acid reduction may only be seen in those with serum uric acid high enough to harm platelet and endothelial function while uric acid reduction in those with lower levels may compromise plasma oxidant activity such that this could be of detriment.

## 10. Summary

There is a now a wealth of epidemiological, animal, and now clinical data to suggest the benefits of strategies to lower uric acid and inhibit xanthine oxidase. Large scale trials with clinical endpoints are justified to address this important question and to define treatment thresholds and targets and to clarify if benefit is real.

## Figures and Tables

**Table 1:** The relationship between serum uric acid and vascular outcomes.

Ref	Population	Change in outcome measure
[5]	Acute stroke	RR 1.27 (1.18–1.36)^a^ for recurrent vascular events
[6]	Acute stroke	Serum uric acid ↑ in those with early clinical deterioration (*P* = .001)
[7]	Acute Stroke	OR 1.37 (1.13–1.67) for early death^b^
[8]	Acute Stroke	OR 1.12 (1–1.25) per additional mg/dL uric acid for good outcome
[7]	Acute Stroke	OR 1.57 (1.02–2.42) for poor outcome*
[9]	Diabetes	HR 1.91 (1.24–2.94) for stroke^b^
[10]	Diabetes and stroke	HR 1.49 (1.21–1.84) for recurrent CV event^a^
[11]	Coronary Disease	HR 1.5 (1.02–2.1) for all cause mortality^b^
[12]	Coronary Disease	HR 1.23 (1.11–1.36) for all cause mortality^c^
[13]	Hypertension	HR 1.32 (1.03–1.69), for CV events^b^
[14]	Hypertension	HR 1.22 (1.11–1.35) for CV disease^d^
[15]	Hypertension	HR 1.14 (1.02–1.27) for CV mortality^e^ HR 1.34 (1.14–1.57) for fatal stroke^e^
[16]	Hypertension	HR 1.73 (1.01–3) for CV event rates^b^
[17]	Hypertension	HR 1.03 (0.93–1.14) for CV mortality^e^ HR 1.06 (0.99–1.13) for all CV events^e^
[18]	Healthy Volunteers	HR 1.16 (*P* < .001) for all-cause mortality^b^ HR 1.35 (*P* = .02) for ischaemic stroke^b^
[19]	Healthy Volunteers	HR 1.35 (1.20–1.52) for CV mortality^b^ HR 1.37 (1.09–1.74) for stroke^b^
[20]	Healthy Volunteers	OR 2.6 (1.2–5.4) for white matter hyperintense signals on MRI imaging^b^

Results expressed as ratio and 95% CI. ^a^per additional 0.1 mmol/L in serum uric acid, ^b^ for highest versus lowest group, ^c,d^per additional 0.6 and 0.86 mmol/L in serum uric acid respectively, ^e^for each 50 *μ*mol/L increment in serum uric acid. *on univaiate analysis. RR—Relative risk, OR—Odds ratio, HR—Hazard ratio, CV—Cardiovascular.
